# Priapism in a 31-Year-Old Male With Paranoid Schizophrenia

**DOI:** 10.7759/cureus.48978

**Published:** 2023-11-17

**Authors:** Eduardo D Espiridion, Sonam Saxena

**Affiliations:** 1 Psychiatry, West Virginia School of Osteopathic Medicine, Lewisburg, USA; 2 Psychiatry, Drexel University College of Medicine, Philadelphia, USA; 3 Psychiatry, Philadelphia College of Osteopathic Medicine, Philadelphia, USA; 4 Psychiatry, Reading Hospital, West Reading, USA

**Keywords:** alpha-1 adrenergic agonist, nonischemic priapism, medication side effects, second-generation antipsychotics, paranoid schizophrenia

## Abstract

Priapism is a painful and emergent side effect that has been linked to some antipsychotics and other psychiatric medications, most often trazodone. This is thought to be due to some level of alpha-1 adrenergic blockade by these medications. Aripiprazole is an atypical antipsychotic with notably weak alpha-1 adrenergic antagonism. Thus, we report on a unique case of aripiprazole-induced priapism in a patient with schizophrenia and recurrent episodes of antipsychotic-induced priapism. This study offers insight into the potential mechanism of aripiprazole-induced priapism and offers alternative medications, such as olanzapine and lumateperone, to treat the patient's ongoing psychotic disorder.

## Introduction

Priapism is a medical emergency; it describes the condition of a persistent (over four hours) and possibly painful erection due to dysregulated detumescence [1]. Priapism can be split into ischemic (low-flow) and nonischemic (high-flow) conditions, with ischemic priapism accounting for around 95% of cases [2]. Ischemic conditions can progress to something as severe as gangrene of the penis. This can be severely painful due to tissue ischemia [1]. Nonischemic conditions are often self-resolving and occur secondary to direct penile or perineal trauma [1].

There is a diverse range of causes of priapism, some of which include medications, such as intracavernous vasoactive injections [3]. Of special interest here is the link to psychotropic medications such as trazodone, topiramate [4], and more. Other causes include sickle cell disease, total parenteral nutrition (TPN) [1], or idiopathic causes [2].

Low-flow priapism is thought to be due to failure of the detumescence mechanism that normally allows outflow of blood from the cavernosa of the penis to relieve erection [3]. This can be disrupted by pharmacologic agents that interfere with the smooth muscle cell (SMC) tone that is controlled via the autonomic nervous system [3]. Low-flow priapism is treated rapidly with increasing outflow of blood via corporal aspiration or medical therapy, in the case of sickle cell anemia [1]. Consequences of untreated low-flow priapism can include penile fibrosis and impotence, as smooth muscle necrosis and endothelial destruction occur within the first 48 hours [1,3].

Whereas low-flow priapism occurs due to decreased venous outflow and vascular stasis, high-flow priapism occurs due to an increased arterial inflow with normal venous outflow. This is thought to be due to the formation of fistulas secondary to the trauma, allowing blood in the arteries to bypass the normal high resistance in the rest of the circuit [3]. High-flow priapism does not often progress to ischemia and thus is often not painful [1]. Treatment of high-flow priapism can include arterial embolization or shunting [5].

Recurrent self-limiting ischemic priapism can also occur, known as stuttering priapism. Treatment includes the same interventions of corporal aspiration, alpha-1 agonist injection, and shunting if necessary [3].

## Case presentation

A 31-year-old male with a past psychiatric history significant for paranoid schizophrenia presented to the emergency department with recurrent episodes of priapism, with this presentation being his seventh occurrence. At the time of presentation, his only scheduled medication included aripiprazole. He explained that his episodes of priapism started more than seven years ago when he tried trazodone, which has since been discontinued. He has experienced recurrent episodes since then, also notably when taking paliperidone. He denied any direct trauma to the penile or perineal areas. His urine analysis showed only positive for marijuana use, which he believes is unrelated to the priapism episodes. He did not endorse any history of hypercoagulable states, including sickle cell anemia, thalassemia, and multiple myeloma. This episode of priapism began in the morning with a sharp pain and culminated in a persistent erection and inability to sleep that night. This was resolved with penile aspiration and lavage of the cavernosa in the emergency department that night. However, the patient experienced another episode of priapism the morning after and returned to the emergency department. This episode was resolved with terbutaline and penile block. Aripiprazole was stopped, and the patient followed up outpatient for further medication management.

The patient has a history of paranoid and persecutory delusions which exacerbated after the priapism episodes. He felt that his providers were intentionally hurting him. He also had a history of psychiatric medication noncompliance which was concerning as he had a documented history of multiple suicide attempts whenever he was floridly psychotic. The patient responded well to reassurance and supportive psychotherapy. He was slated to follow-up at a local mental health center the day after discharge to explore other treatment strategies for paranoid schizophrenia. There were no residual pain complaints on the day of the hospital discharge.

## Discussion

Most reports of antipsychotic-induced priapism are of the ischemic kind. Antipsychotics are thought to induce priapism due to their alpha-1 adrenergic receptor blockade [4,6]. This allows parasympathetic activity to dominate and allows persistent vasodilation and subsequent erection of the penis. Of the atypical antipsychotics, however, aripiprazole is thought to have the weakest affinity for these receptors, making this an unexpected and unique case of medication-induced ischemic priapism [7].

There has been another reported case of aripiprazole-induced priapism, onset within seven hours of taking his first 10 mg dose [6]. This case was resolved with a single 2 mL injection of adrenaline and required no repeat treatment. This is different, however, from the current case because that paper's described patient had no record of similar previous episodes and was switched to amisulpride, a D2 dopamine receptor antagonist, without issue. Given the repeated bouts of penile trauma and low likelihood of aripiprazole causing alpha-1 adrenergic blockade, there was an initial suspicion of nonischemic priapism. However, upon review, the clinical presentation aligned more with ischemic priapism given the painful episode, administration of an alpha-1 adrenergic agonist (although weak), and resolution with aspiration of the cavernosa. It should be noted that the first episode of priapism was also noted to be ischemic priapism due to trazodone; since then, he has experienced recurrent priapism with typical and atypical antipsychotics. A case of conversion from low-flow priapism to high-flow priapism has also been reported by Lutz et al. [8], but this is unlikely given the clinical picture fitting more with a rare case of aripiprazole-induced ischemic priapism. Given the repeated episodes of ischemic priapism, a case of stuttering priapism was also considered. However, episodes of stuttering priapism are often self-limited and resolve within a few hours [2]. In this patient's case, the priapism only resolved after aspiration, which was after a period of time longer than three hours. Per this patient's history, none of his previous episodes self-resolved. Again, this case more likely fit the case of medication-induced ischemic priapism.

The consequences of ischemic priapism differ from those of nonischemic priapism. Ischemic priapism is characterized by a reduction in intracavernous blood flow and can lead to irreversible ischemic tissue damage within six hours [2]. In terms of future management, this patient will still require antipsychotic treatment due to his recurrent and active psychosis and schizophrenia. Even upon discharge, the patient was still endorsing delusions of grandeur. As his trials with antipsychotics, typical and atypical, have shown recurrent episodes of priapism likely due to their alpha-1 antagonism, it is reasonable to try a different drug class altogether. Olanzapine is a serotonin antagonist at 5-HT2A, 5-HT2B, 5-HT2C, alpha-1 adrenergic, and H1 histaminergic receptors that has been administered as an antipsychotic to people with schizophrenia [9]. This may also be an appropriate choice for the further management of psychotic symptoms. This patient does not have a medical history including any of the following: high BMI, metabolic syndrome, hyperlipidemia, or cardiovascular disease. For management of patients with these risk factors, given olanzapine's risk of developing weight gain and metabolic syndrome, a combination of olanzapine and lumateperone may be trialed [10]. Lumateperone is a D2 and 5-HT2A receptor agonist, against dopamine and serotonin, respectively, but is considered higher-affinity for the D2 receptor than other antipsychotics, such as risperidone and olanzapine [8]. This may allow a lower incidence of extrapyramidal side effects (EPS) due to increased D2 receptor blocking [8], providing an improved side effect profile compared to other antipsychotics. Lumateperone is also considered to have a favorable side effect profile with its minimal risk of weight gain and minimal metabolic adverse effects.

In terms of administration methods, atypical depot injections can be considered, given his history of medication noncompliance. In addition, the use of injectable depots makes it more difficult to halt medication-induced priapism in the case of recurrence.

## Conclusions

This case serves as an important addition to the scarce literature about aripiprazole-induced priapism. This patient has been experiencing recurrent episodes of priapism linked to his psychotropic medication use, including episodes due to trazodone, paliperidone, and now aripiprazole. Future considerations for this patient must include alternative medications that target NMDA receptors or serotonin receptors.
